# Isthmin 1, matrix metalloproteinase 8 and asprosin as potential biomarkers in periodontitis with obesity

**DOI:** 10.1186/s12903-025-06868-5

**Published:** 2025-10-08

**Authors:** Yuwei Zhang, Yue Jia, Shanmei Zhao, Yanqi Wang, MaErWa MuLaTiHaJi, Xiao Luo, Ru Jia

**Affiliations:** 1 Key Laboratory of Shaanxi Province for Craniofacial Precision Medicine Research, College of Stomatology, Xi’an Jiaotong University, 98 Xiwu Road, Xi’an, Shaanxi Province 710004 China; 2 Clinical Research Center of Shaanxi Province for Dental and Maxillofacial Diseases, Xi’an, Shaanxi Province 710004 China; 3 Department of Biomedical Engineering, The Chinese University of Hong Kong, Hong Kong SAR, 999077 P. R. China; 4 Department of Digital Oral Implantology and Prosthodontics, College of Stomatology, Xi’an Jiaotong University, Xi’an, 710004 Shaanxi Province China; 5 Department of Physiology and Pathophysiology, School of Basic Medical Sciences, Xi’an Jiaotong University Health Science Center, Xi’an, 710061 Shaanxi China

**Keywords:** Periodontitis, Obesity, Isthmin 1, Matrix metalloproteinase 8, Asprosin

## Abstract

**Objective:**

Isthmin 1 (ISM1), matrix metalloproteinase 8 (MMP-8) and asprosin are cytokines involved in the inflammatory and obesity. But the role of them in the periodontitis with obesity remains unknown. This study aims to determine the levels of ISM1, MMP8 and asprosin from gingival crevicular fluid (GCF), saliva and plasma in patients with obesity and periodontitis, and then analyze the association between them and periodontitis with obesity.Materials and methods: A total of 60 patients were divided into four groups based on body mass index (BMI), clinical probing depth (PD), clinical attachment level (CAL), and panoramic photography. The groups were (1) normal weight & periodontally healthy participants (NnP), (2) obese & periodontally healthy participants (OnP), (3) normal weight participants with periodontitis (NP) and (4) obese participants with periodontitis (OP). The GCF, saliva and plasma levels of human ISM1, MMP-8 and asprosin were quantified by Enzyme-Linked Immunosorbent Assay.Results: The levels of ISM1 and MMP-8 in plasma, which were positively associated with BMI, were higher in two obese groups as compared with non-obese groups with significant difference. MMP-8 and ISM1 were also positively correlated with pocket depth among whole population. Based on negative binomial regression, groups of obesity saw a significant positive correlation between CAL/PD and ISM1, MMP-8, asprosin in GCF, as well as BMI.

**Conclusion:**

In conclusion, circulating levels of ISM1 and MMP-8 were significantly elevated in patients with obesity and positively correlated with BMI. Importantly, our study demonstrates that ISM1 and MMP-8 levels in GCF reflect the severity of periodontitis, as evidenced by their strong positive association with clinical parameters such as PD and CAL. Non-invasive method of testing ISM1, MMP-8 and asprosin could be an effective reference tool for evaluating periodontitis in patients with obesity. All in all, ISM1, MMP-8 and asprosin might be potential biomarkers in periodontitis with obesity.

**Supplementary Information:**

The online version contains supplementary material available at 10.1186/s12903-025-06868-5.

## Introduction

Accumulated evidence from clinical trials, experimental studies and systematic reviews has presented a significant association between periodontitis/tooth loss and obesity, both in terms of a higher prevalence of periodontitis is accompanied with a higher BMI among subjects with both overweight and obese, as well as severer periodontitis as compared with matched controls. This association has been explained through the systemic, periodontal inflammation and bone loss elicited by the comorbidity effect [1–5].

Clinical and experimental studies with subjects of obesity and periodontitis have explained some potential mechanisms [6–8], especially the role of adipokines in the process of excessive adipose tissue and periodontitis [9–12]. However, a more defining pathogenic pathway still needs to be identified for further diagnose, prognose and treatment of the comorbidity. Matrix metalloproteinase (MMP)−8 as macromolecules secreted by neutrophils in extracellular matrix, any imbalance between the MMPs and tissue inhibitors initiates the destruction of collagen in periodontal tissue, resulting in chronic periodontitis [13]. Isthmin 1 (ISM1), as a novel secreted adipokine or as a regulator of lymphocyte effector function that initially identified as promoting glucose uptake and improving hepatic steatosis as well as glucose tolerance and may get involved in innate and adaptive immune responses [14, 15]. It is found to be able to suppress inflammation during infection [16, 17] and highly express both in childhood and adulthood obesity as compared with peer control [14, 18]. Furthermore, asprosin, as described to participant in process of lipid and bone metabolism, together with glucose regulation, may also associates with periodontitis and obesity [6]. Nevertheless, it is ill-illustrated that the role of MMP-8, ISM1 and asprosin as a co-morbidity effect in chronic periodontitis and obesity in the real-world clinical practice. Therefore, the purpose of the present clinical investigation is focus on the impact of participants with obesity and periodontitis on both invasive circulating level and non-invasive (GCF and saliva) levels of these selected biomarkers. In this context, our study aimed to correlate levels of MMP-8/ISM1/asprosin and periodontal/obesity parameters to determine potential biomarkers for the early diagnosis, prognose, risk assessment and treatment of patients with chronic periodontitis and obesity.

## Materials and methods

A consecutive recruitment of 60 adult patients for the present cross-sectional study was conducted from September 2023 to May 2024 at Stomatology hospital of Xi’an Jiaotong University, and the whole process was in full accordance with ethical principles. Body mass index (BMI) of each participant, as an indicator of obesity, was calculated and classified according to the cut-off value of Western Pacific Regional Office of World Health Organization for obesity in adult Asians [19, 20] (normal weight: 25 > BMI > 18.5 kg/m^2^; obesity: BMI ≥ 25 kg/m^2^). There were four groups with each 15 participants according to the minimum sample size calculation: $\:n=\frac{{Z}_{1-\frac{\alpha\:}{2}}^{2}\times\:p\times\:(1-p)}{{\text{d}}^{2}}$, (study power: 85%, 95% confidence level (CI), and a prevalence rate of periodontitis in the groups of normal weight and obesity as described in [4, 5, 7]). Groups were presented as normal weight & periodontally healthy participants (NnP); obesity & periodontally healthy participants (OnP); normal weight participants with periodontitis (NP) and participants with obesity and periodontitis (OP). There were 32 males and 28 females, aged from 26 to 50 years. Patients taking orthodontic treatments, periodontal treatments within the past 6 months, lipid-lowering therapy, immunosuppressant therapy, antibiotics and non-steroid anti-inflammatory drugs within the past 3 months or having any of the known effects of periodontal health status, including diabetics, metabolic syndrome, hypertension, tumors, pregnancy and lactation were excluded. Also, heavy smokers (more than 20 cigarettes/day) and patients left with less than 10 teeth were removed. The questionnaires for the integrated medical and lifestyle assessment form (including demographics & medical history, smoking behavior survey, and alcohol consumption survey) were provided in Supplementary Table S1.

The protocol of clinical trial conformed to the 1964 declaration of Helsinki and subsequent amendments, and was approved by the Ethical Committee and Review Board of the Stomatology hospital of Xi’an Jiaotong University. The written informed consent was obtained prior to clinical periodontal examination, intraoral GCF and saliva, as well as intravenous blood collection.

### Clinical periodontal assessments

All clinical periodontal measurements were followed the latest definition of periodontitis based on the 2017 World Workshop [5], and was diagnosed when met at least one of following criteria: interdental clinical attachment loss level (CAL) not less than 2 non-adjacent teeth; buccal or oral CAL not less than 3 mm with periodontal pocket depth (PD) more than 3 mm at over 2 teeth [21].

A well-trained periodontist performed all the periodontal examinations. Full-mouth clinical periodontal assessments of all remaining teeth (without the third molars) at six-point for each tooth (mesio-buccal, buccal, disto-buccal, disto-lingual, lingual and mesio-lingual), including PD, CAL, plaque index (PI) and bleeding upon probing (BOP, +/-) were recorded after the oral examination. And bone loss was captured via panoramic radiograph for auxiliary evaluation of alveolar bone resorption levels.

### GCF, saliva and plasma samples collection

The GCF collection sites were determined based on a comprehensive clinical assessment, excluding gingivitis cases. GCF was sampled from the sites with the deepest periodontal pocket depth (as determined by PD measurements) by placing the filter paper strips (Whatman 30#, OCOME, Hangzhou, China) gently at the entrance of the gingival sulcus for 30 s. Meanwhile, saliva was collected by placing the filter paper strips under the tongue for 30 s. The strips were stored in dry microcentrifuge tubes.

Afterwards, the microcentrifuge tubes were weighed. The concentration of 0.15 mg:150µL was set as 1 for the GCF sample, while the concentration of 0.30 mg:150µL was set as 1 for the saliva sample, and a total of 150 µL buffer sample solution was added for dilution. Then, the solution was centrifuged (1500 rpm/min, 4 °C, 15 min), and supernatant was extracted and stored in a refrigerator at −80 °C for subsequent usage.

After 12 h of fasting, patients were extracted with 3.5 mL venous blood restoring in a vacuum tube, then 2 mL of the sample was centrifuged (3000 rpm/min, 4 °C, 15 min) for biochemical screening tests. The plasma was separated and temporally stored at −20 °C until examination.

### Biochemical analysis

The biochemical analysis was performed on all samples from 60 participants. Fasting blood glucose (FBG) was determined using OneTouch Sure Step Test Strips (Johnson &Johnson, New Brunswick, NJ, USA) as soon as the venous blood samples were taken. GCF and blood/plasma to be tested were balanced at room temperature for 15 min. Plasma total cholesterol, triglycerides, high-density lipoprotein-cholesterol (HDL-C) and low-density lipoprotein-cholesterol (LDL-C) were measured by the corresponding assay kits (A111-1-1, A110-1-1, A112-1-1 and A113-1-1; Nanjing Jiancheng Bioengineering Institute, Nanjing, China).

Concentrations of MMP-8, ISM1 and asprosin in the biofluids were measured using the matched human Enzyme-Linked Immunosorbent Assay (ELISA) kits (MMP-8: U96-1564E, YOBIBIO, Shanghai, China; ISM1: EH4520, FineTest, Wuhan, China; asprosin, EH4176, FineTest, Wuhan, China) and the plates were read at 450 nm by a Microplate Reader (BioRad Laboratories, Hercules, CA, USA), respectively. The examiner performed all the process and recorded the results while blinded to the case-control status.

### Statistical analysis

All the evaluated parameters displayed in the tables and figures were presented as mean ± standard deviation (SD). Normality was confirmed through the Shapiro–Wilk normality test (*n* ≤ 50 per group), then the corresponding statistical approach was selected. Comparisons among multiple groups were evaluated by one-way analysis of variance (one-way ANOVA) or nonparametric test (Kruskal-Wallis H test) and the statistical significances between groups were compared with the Fisher post hoc least significant difference (LSD) test. Spearman’s correlation coefficient was performed for correlation analysis. Negative binomial regression models were employed to address overdispersion (variance > mean) in the count data and to examine the association between clinical periodontal parameters (PD and CAL) and various factors in participants with or without obesity. SPSS version 22 (SPSS Inc, Chicago, IL, USA) and GraphPad Prism Software version 9.4.0 (GraphPad Software, La Jolla, CA, USA) were used for the statistical analyses in this study. The values of *p* < 0.05 (two-tailed) represented differences between groups were statistically significant.

## Results

### General information and lipid metabolism of subjects

**Fig. 1:**
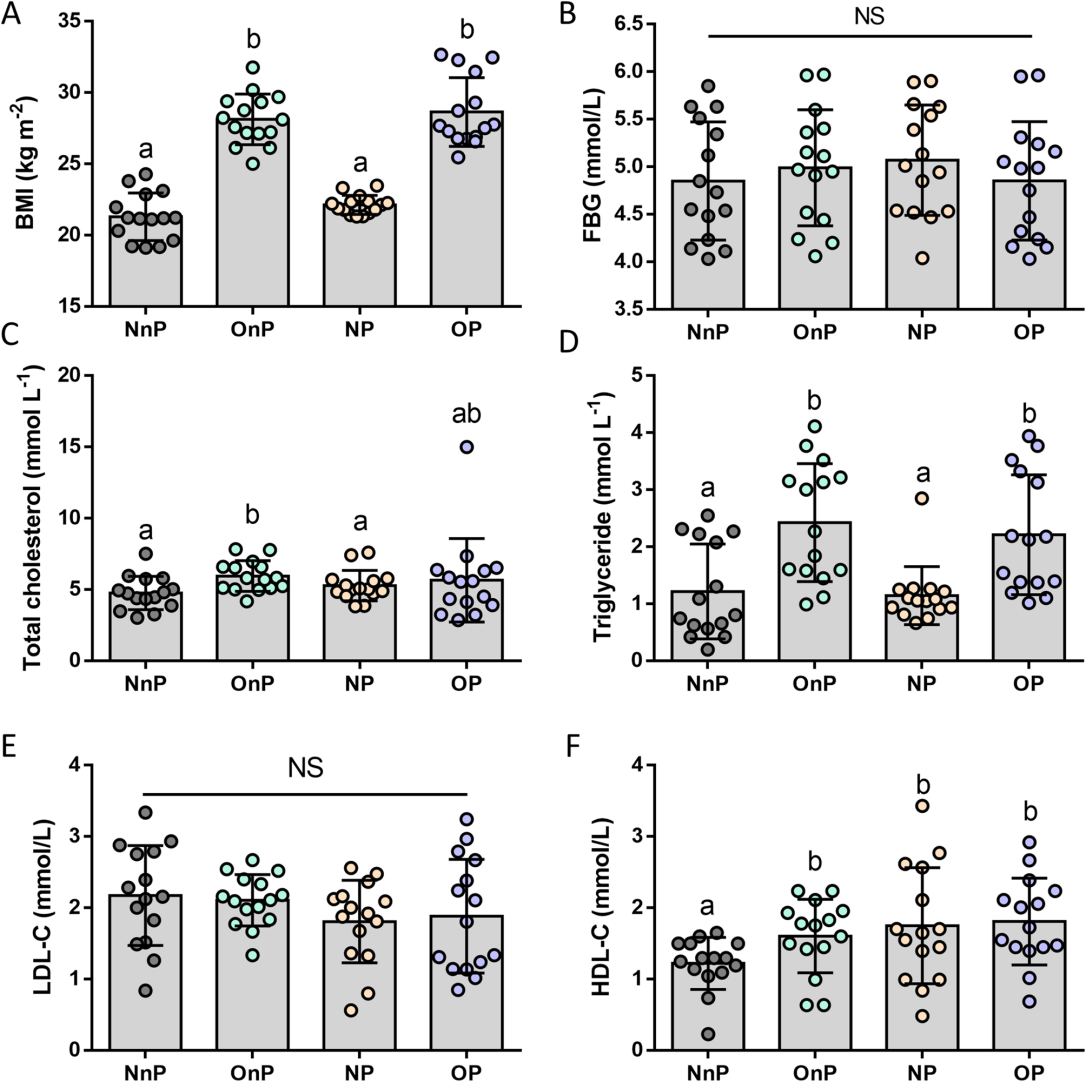
Status of obesity and lipid metabolism level in plasma. Data were presented as mean ± SD, *n* = 15. Different letters indicated statistical differences between two groups. BMI, body mass index; FBG, fasting blood glucose; LDL-C, low-density lipoprotein-cholesterol; HDL-C, high-density lipoprotein-cholesterol, NnP, normal weight & periodontally healthy participants; OnP, obese & periodontally healthy participants; NP, normal weight participants with periodontitis and OP, obese participants with periodontitis. *P* value of between two groups confirmed by LSD test or nonparametric test from Fig. 1A and F was displayed in Table S2

As described in Table 1, there was no significant difference in age distribution (*p* = 0.762). As consistent with the initial exclusion criteria: no participant suffered from hypertension, metabolic disease and immune disease, neither pulmonary disease nor renal disease. And significant difference in the obesity-indicator (BMI) was discovered between obese and non-obese groups (Table 1; Fig. 1A), indicating that the grouping of obesity and non-obesity was reasonable and the experimental data had high comparability. Biochemical analysis (Table 2; Fig. 1B-F, and Table S2) suggested that patients with diabetes were excluded from the study, and triglyceride levels of obese groups were significantly higher than non-obese groups.

**Table 1:** Demographic analysis of the study groups (*n* = 60)

Study group	Group 1 NnP	Group 2 OnP	Group 3 NP	Group 4 OP	*P* value
N	15	15	15	15	-
Age (years)	37.93 ± 6.29	39.48 ± 9.53	41.49 ± 7.72	41.81 ± 9.20	0.762
Gender (male/female)	8/7	8/7	7/8	9/6	-
Hypertension (yes, no)	0/15	0/15	0/15	0/15	-
Pulmonary disease (yes, no)	0/15	0/15	0/15	0/15	-
Cardiovascular disease (yes, no)	0/15	0/15	0/15	1/14	-
Renal disease (yes, no)	0/15	0/15	0/15	0/15	-
Metabolic disease (yes, no)	0/15	0/15	0/15	0/15	-
Immune system disease (yes, no)	0/15	0/15	0/15	0/15	-
Current alcohol habit (yes, no)	3/12	3/12	5/10	4/11	-
Current smoking habit (yes, no)	4/11	3/12	7/8	6/9	-
Physical activity practice (yes, no)	5/10	4/11	4/11	2/13	-
Frequency of brushing (More/Not more than once/day)	15/0	14/1	11/4	10/5	-

All quantitative data were presented as mean ± SD. One-way ANOVA or Kruskal-Wallis H test was used as the statistically test

**Table 2:** Biochemical analysis of the study groups (*n* = 60)

Study group	Group 1 NnP	Group 2 OnP	Group 3 NP	Group 4 OP	*P* value
N	15	15	15	15	-
BMI (kg/m^2^)	21.29 ± 1.66^a^	28.12 ± 1.78^b^	22.13 ± 0.67^a^	28.63 ± 2.41^b^	0.000*
Fasting blood glucose (FBG, mmol/L)	4.85 ± 0.62	4.99 ± 0.61	5.07 ± 0.58	4.85 ± 0.62	0.699
FBG > 6.1 mmol/L	0	0	0	0	-
Total cholesterol (mmol/L)	4.76 ± 1.17	5.95 ± 1.07	5.27 ± 1.07	5.65 ± 2.92	0.282
Triglycerides (mmol/L)	1.22 ± 0.83^a^	2.42 ± 1.03^b^	1.14 ± 0.51^a^	2.21 ± 1.05^b^	0.000*
HDL-C (mmol/L)	1.22 ± 0.36^a^	1.60 ± 0.51^b^	1.75 ± 0.81^b^	1.81 ± 0.61^b^	0.041*
LDL-C (mmol/L)	2.17 ± 0.70	2.11 ± 0.36	1.81 ± 0.58	1.88 ± 0.80	0.336

All quantitative data were presented as mean ± SD. One-way ANOVA or Kruskal-Wallis H test was used as the statistically test*. Different letters (a, b) indicated statistical differences among groups confirmed by LSD test or nonparametric test. *indicated a statistically significant difference at the *P*<0.05 level

### Clinical periodontal measurements

After thorough clinical periodontal measurements, periodontitis-related clinical indicators: PI, PD, CAL, and BOP (+/-) of each site/patient were carefully recorded. PI of patients with periodontitis were significantly higher than those without (NP: 3.84 ± 0.92, OP: 3.67 ± 1.48 vs. NnP: 1.53 ± 0.66, OnP: 1.82 ± 0.89, *p* = 0.000). Also, PD level (mm) of patients with periodontitis were significantly higher than those without (NP: 5.53 ± 1.60, OP: 5.39 ± 1.31 vs. NnP: 1.68 ± 0.39, OnP: 1.66 ± 0.20, *p* = 0.000). And obese participants with periodontitis had a statistically significant higher level of CAL (5.27 ± 1.88 mm) than normal weight participants with periodontitis (4.77 ± 2.03, *p* = 0.000). Moreover, not less than 1/2 of patients with periodontitis were ascertained as positive of bleeding upon probing in more than 50% sites of the remaining teeth (Table 3).

**Table 3:** Periodontal status of the study groups (*n* = 60)

Study group	Group 1 NnP	Group 2 OnP	Group 3 NP	Group 4 OP	*P* value
N	15	15	15	15	-
PI	1.53 ± 0.66^a^	1.82 ± 0.89^a^	3.84 ± 0.92^b^	3.67 ± 1.48^b^	0.000*
PD (mm)	1.68 ± 0.39^a^	1.66 ± 0.20^a^	5.53 ± 1.60^b^	5.39 ± 1.31^b^	0.000*
CAL (mm)	-	-	4.77 ± 2.03^a^	5.27 ± 1.88^b^	0.000*
BOP (Positive in more than 50% remaining teeth)	0/15	0/15	5/10	7/8	-

All quantitative data were presented as mean ± SD. One-way ANOVA or Kruskal-Wallis H test was used as the statistically test. Different letters (a, b) indicated statistical differences among groups confirmed by LSD test or nonparametric test. *indicated a statistically significant difference at the *P*<0.05 level

### Concentration of ISM1, MMP-8 and asprosin in biofluids

**Fig. 2:**
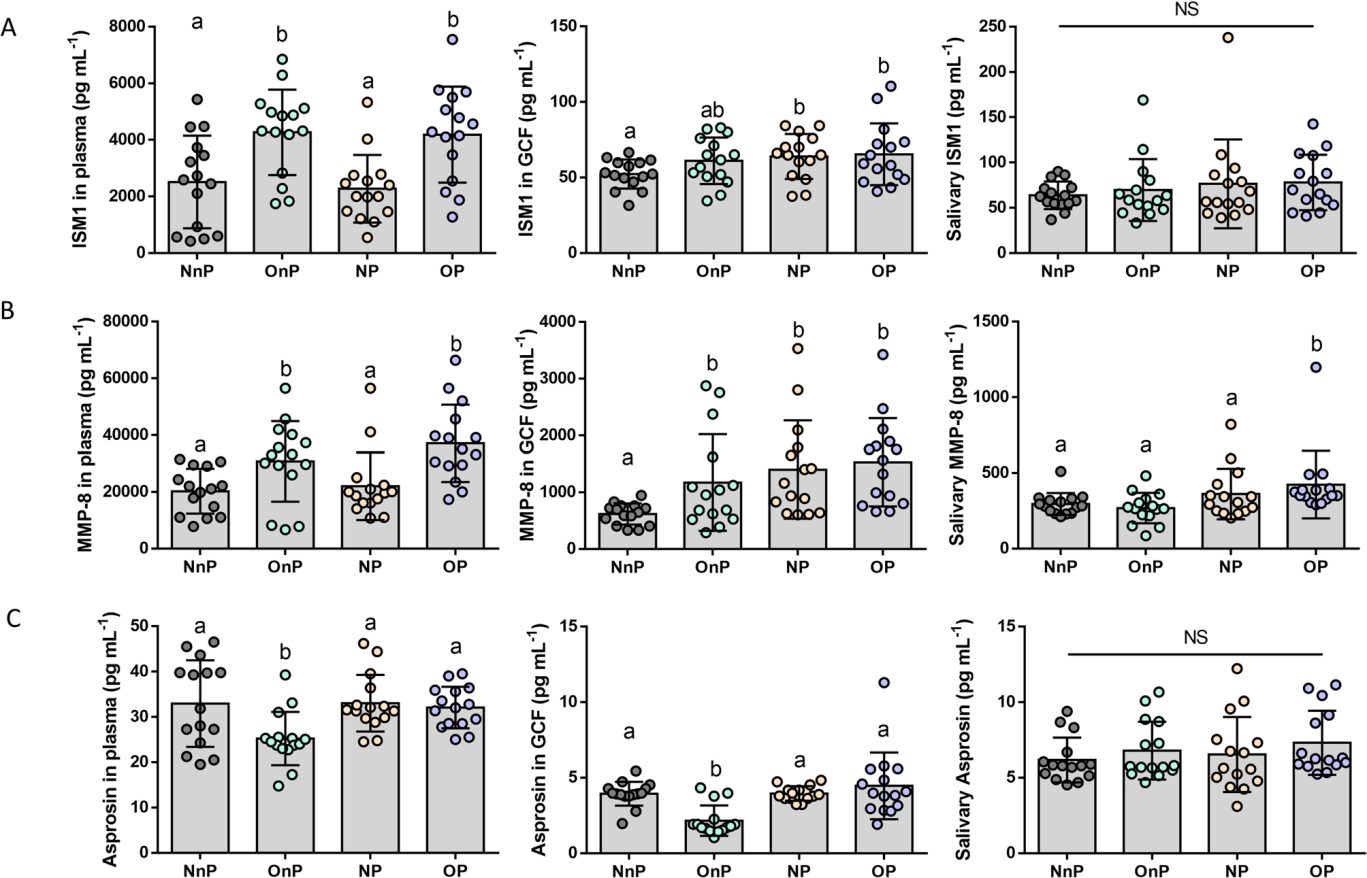
Concentrations of adipokines/cytokines obtained in plasma, GCF and saliva of the studied groups were measured by ELISA. Cytokines including ISM1 (**A**), MMP-8 (**B**) and asprosin (**C**). Data were presented as mean ± SD, *n* = 15. Different letters indicated statistically significant differences between two groups. ISM1, Isthmin 1; MMP-8, matrix metalloproteinase 8; NnP, normal weight & periodontally healthy participants; OnP, obese & periodontally healthy participants; NP, normal weight participants with periodontitis and OP, obese participants with periodontitis. *P* value of between two groups confirmed by LSD test or nonparametric test from Fig. 2A and C was displayed in Table S3

The results showed that the levels of ISM1 and MMP-8 in plasma were higher in two obese groups as compared with non-obese groups with significant difference (Table 4; Fig. 2A and B), while groups of periodontitis saw a significant higher ISM1 level in GCF (Table 4; Fig. 2A). GCF MMP-8 level was increased in the other three groups than the NnP group, while salivary MMP-8 level only elevated in group of OP (Table 4; Fig. 2B). Interestingly, we found level of asprosin in plasma and GCF were only decreased in OnP as compared with other three groups (Table 4; Fig. 2C, and Table S3).

**Table 4:** Biochemical analysis of the study groups (*n* = 60)

Study group	Group 1 NnP	Group 2 OnP	Group 3 NP	Group 4 OP	*P* value
N	15	15	15	15	-
ISM1 plasma (pg/mL)	2511.74 ± 1637.52^a^	4268.60 ± 1511.24^b^	2274.49 ± 1195.21^a^	4185.78 ± 1694.48^b^	0.000*
ISM1 GCF (pg/mL)	52.32 ± 9.65^a^	61.06 ± 15.37^ab^	63.91 ± 14.87^b^	65.34 ± 20.46^b^	0.109
ISM1 saliva (pg/mL)	63.81 ± 15.17	69.65 ± 34.22	76.48 ± 49.08	78.03 ± 30.62	0.654
MMP-8 plasma (pg/mL)	20796.85 ± 7494.89^a^	30752.23 ± 14175.06^b^	22001.97 ± 11912.44^a^	37114.44 ± 13597.50^b^	0.001*
MMP-8 GCF (pg/mL)	618.50 ± 185.79^a^	1171.21 ± 850.82^b^	1399.26 ± 865.87^b^	1528.13 ± 776.30^b^	0.006*
MMP-8 saliva (pg/mL)	295.98 ± 72.74^a^	268.45 ± 100.46^a^	361.71 ± 166.75^a^	405.93 ± 229.7 ^b^	0.028*
Asprosin plasma (pg/mL)	32.93 ± 9.53^a^	25.18 ± 5.94^b^	31.44 ± 9.38^a^	32.05 ± 4.60^a^	0.031*
Asprosin GCF (pg/mL)	3.94 ± 0.79^a^	2.16 ± 1.01^b^	3.96 ± 0.48^a^	4.47 ± 2.21^a^	0.000*
Asprosin saliva (pg/mL)	6.18 ± 1.48	6.80 ± 1.91	6.54 ± 2.48	7.31 ± 2.12	0.269

All quantitative data were presented as mean ± SD. One-way ANOVA or Kruskal-Wallis H test was used as the statistically test. Different letters (a, b) indicated statistical differences among groups confirmed by LSD test or nonparametric test. *indicated a statistically significant difference at the *P*<0.05 level

### Correlation analyses

#### Correlation in whole population

Through correlation analysis, obesity-related clinical indicator (BMI) was positively associated with plasma levels of triglycerides and total cholesterol in the included participants. Higher BMI value was correlated with increased plasma ISM1 level, and plasma or GCF level of MMP-8.

**Fig. 3:**
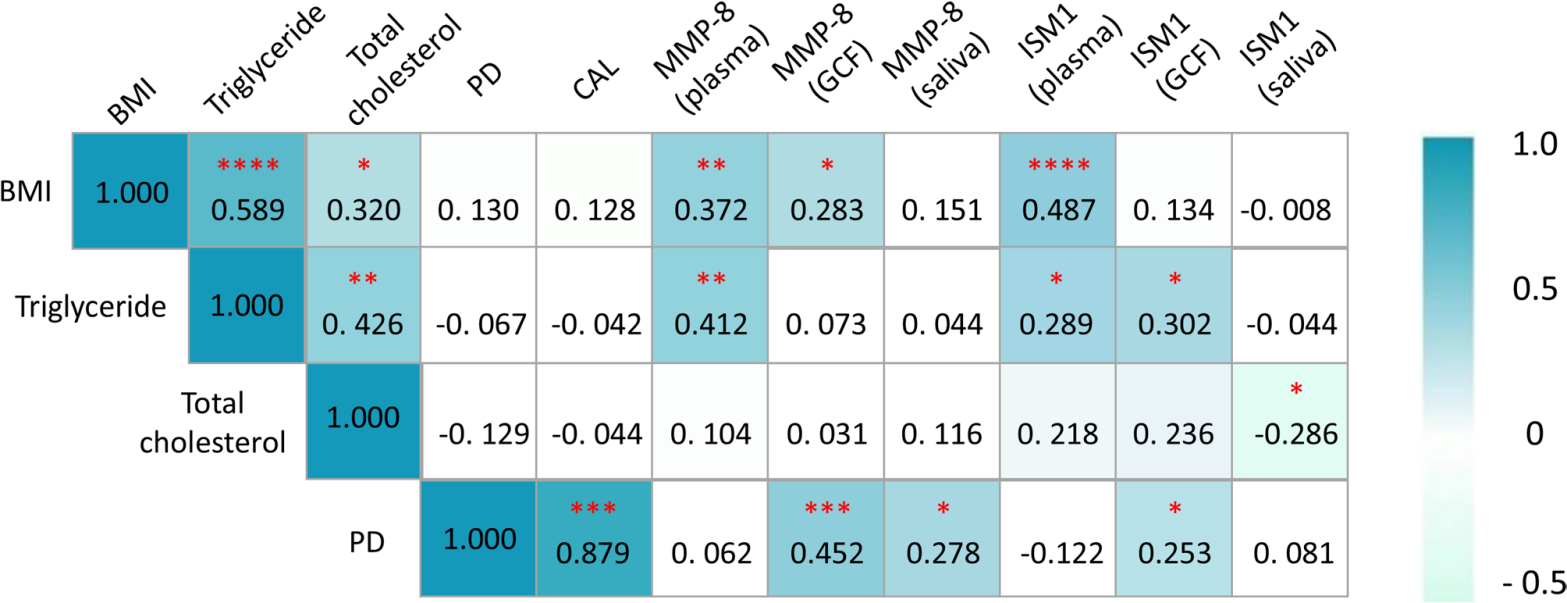
Correlation analysis of obesity-related and periodontitis-related clinical indicators and biomarkers in whole population (*n* = 60), the Spearman correlation analysis was used as the statistical test. *p < 0.05, **p < 0.01, ***p < 0.001, ****p < 0.0001

Meanwhile, circulating triglyceride level was positively associated with ISM1 level (in plasma or GCF) and MMP-8 in plasma. And higher total cholesterol level was accompanied by a decreased salivary ISM1 level (Fig. 3).

As for the correlation between selected biomarkers in our study, we found MMP-8 in GCF was significantly negatively correlated with salivary ISM1 (Fig S1A), while the salivary and GCF level of MMP-8 showed a synchronous trend (Fig S1B). And there was a positive association between plasma level of MMP-8 and ISM1 (Fig S1C).

Through correlation analysis of periodontal clinical indicators and certain biomarkers, it was found PD was positively correlated with ISM1 and MMP-8 levels in GCF (*r* = 0.2527, *p* < 0.05; *r* = 0.4520, *p* < 0.0005), and salivary MMP-8 level (*r* = 0.2783, *p* < 0.05, Fig. 3).

#### Correlation in participants with obesity

Subsequently, focused population was narrowed into participants with obesity to determine whether the comorbid status influence the degree of periodontitis.

**Fig. 4:**
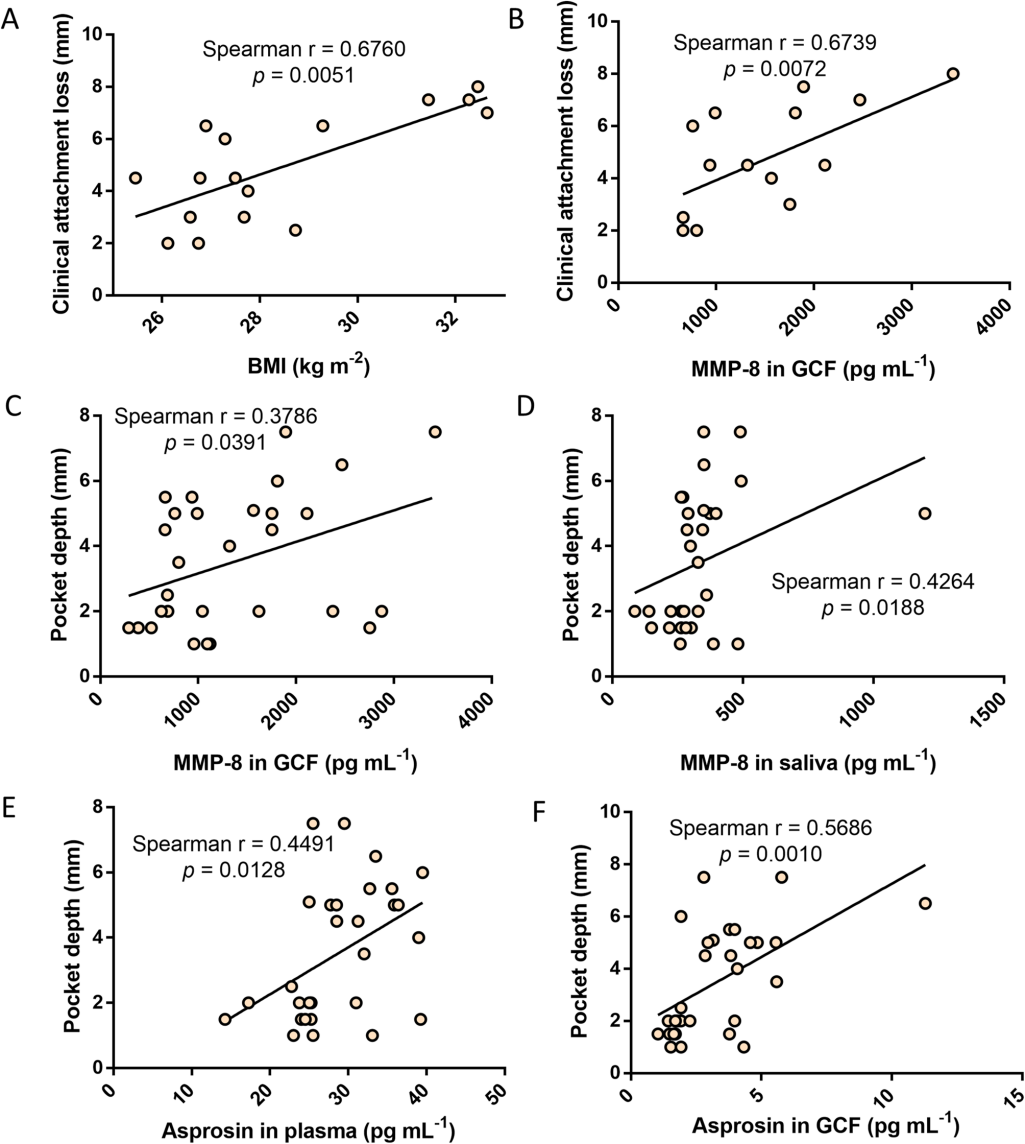
Correlation analysis of clinical indicators and aimed biomarkers in population with obesity, the Spearman correlation analysis was used as the statistical test

BMI and MMP-8 in GCF were found to be positively correlated with CAL level, while, PD was associated with concentration of MMP-8 in GCF/saliva, and circulating/GCF level of asprosin (Fig. 4A-F). These findings indicated aforementioned biomarkers would be effective in revealing the severity of periodontitis in patients with obesity.

### Negative binomial regression model of participants with or without obesity

**Fig. 5:**
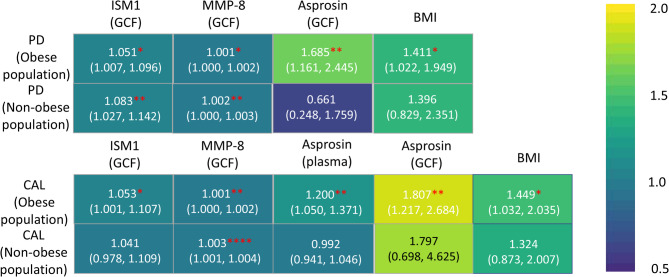
Negative binomial regression analysis of clinical indicators and aimed biomarkers in population with or without obesity, reporting incidence rate ratios (IRRs) with 95% confidence intervals. *p < 0.05, **p < 0.01, ***p < 0.001, ****p < 0.0001

The negative binomial regression analysis revealed significant associations between biomarkers and periodontal outcomes, stratified by obesity status (Fig. 5). Among obese individuals, PD showed significant associations with ISM1 [GCF: Incidence Rate Ratio (IRR) = 1.051, 95%CI = 1.007–1.096], MMP-8 (GCF: IRR = 1.001, 95%CI = 1.000-1.002), asprosin (GCF: IRR = 1.685, 95%CI = 1.161–2.445), and BMI (IRR = 1.411, 95%CI = 1.022–1.949, Table S4). In non-obese individuals, only ISM1 (IRR = 1.083, 95%CI = 1.027–1.142) and MMP-8 (IRR = 1.002, 95%CI = 1.000-1.003) were significantly associated with PD (Table S5). For CAL, obese individuals demonstrated significant associations with ISM1 (GCF: IRR = 1.053, 95%CI = 1.001–1.107), MMP-8 (GCF: IRR = 1.001, 95%CI = 1.000-1.002), asprosin (plasma: IRR = 1.200, 95%CI = 1.050–1.371; GCF: IRR = 1.807, 95%CI = 1.217–2.684), and BMI (IRR = 1.449, 95%CI = 1.032–2.035, Table S6), while non-obese individuals only showed significance for MMP-8 (GCF: IRR = 1.003, 95%CI = 1.001–1.004, Table S7). These findings suggest obesity may modify the influence of biomarkers on periodontal health, with asprosin and BMI exhibiting stronger effects in obese individuals.

## Discussion

The present study focused on the role of MMP-8, ISM1 and asprosin in the process of periodontitis and obesity. As a cross-sectional study, we enrolled a total of 60 consecutive patients for periodontal and physical examinations. Grouping of obesity was based on the gold standard of BMI as well as the assistance of plasm lipid metabolism level. Level of triglycerides and BMI was consistently elevated in groups of obesity than in non-obesity groups. Subsequently, according to the latest 2017 World Workshop, periodontal status, especially CAL, PI and BOP were taken into account for the grading and staging system of diagnose of periodontitis. Therefore, a successful obesity/periodontitis-grouping strategy was established for further assessments.

Interestingly, as shown in Fig. 2, plasma levels of ISM1 and MMP-8 were elevated in individuals with obesity (with or without periodontitis) and positively correlated with BMI. This suggests that higher circulating levels of ISM1 and MMP-8 may reflect obesity-related systemic inflammation, reinforcing obesity as a significant comorbidity in periodontitis. These findings position ISM1 and MMP-8 as potential biomarkers for assessing obesity-driven inflammatory states, even at low-grade systemic levels [22].

Moreover, ISM1 in GCF merely elevated in the groups of periodontitis, we surmised that local/periodontal expression of ISM1 in GCF was increased mostly attributed to the periodontal diseases, in other words, the local periodontal inflammation. However, as confirmed in the correlation analysis, a positive association was found between ISM1 in GCF and PD among whole population, which means whether in healthy or non-healthy groups, PD value was associated with ISM1 in GCF, and such non-invasive evaluation of ISM1 might be the adjunctive tool for the diagnose and prognose of periodontitis. ISM1, which is a secreted adipokine that has dual roles in promoting adipocyte glucose uptake while suppressing hepatic lipid synthesis and lowing lipid accumulation, thus has potential in treating diabetes and fatty liver disease, simultaneously [14]. In the present study, we found that ISM1 in GCF and plasma level was also significantly associated with triglycerides in a positive trend, while salivary ISM1 was negatively associated with total cholesterol, as ISM1 involves in lipid-metabolism in a complex way. Meanwhile, as an anti-inflammatory cytokine, ISM1 had been proved to suppress inflammation during infection [16, 17]. These provoked the insights that elevated circulating ISM1 in our study mainly aimed to against the chronic low-systemic inflammation in obesity accelerated by hypertrophic adipocytes and adipose tissue-resident immune cells, which was also consistent with the former clinical study in human or childhood obesity [14, 18, 23]. Moreover, the higher level of ISM1 in deeper pocket depth, revealing a reconstruction of delicate inflammation balance during the chronic disease. These findings accumulated evidence for understanding the role of ISM1 in the process of inflammation, especially the local and systemic inflammatory status represented by periodontitis and obesity.

As an essential and classic pro-inflammatory cytokine, GCF MMP-8 was significantly increased in any of the recognized situation of inflammation without NnP, while salivary MMP-8 solely increased in OP with an extensive secretion aggravated by dual periodontitis and obesity. MMP-8 (GCF and saliva) was also positively correlated with pocket depth among whole population; and positively correlated with CAL among patients with periodontitis. MMP family is the macromolecules/collagenases in periodontal extracellular matrix (represented by type I collagen) that belongs to zinc-dependent endopeptidases [24]. Except for as one of the most promising biomarkers for periodontitis in oral fluids, MMP-8 was also correlated with BMI and triglycerides, these findings illustrated how this protein might be of special relevance in chairside testing as a novel biomarker of both obesity and periodontitis.

As our former study confirmed the specific role of asprosin in periodontitis among obese rats [6], we found that circulating and GCF level of asprosin decreased in OnP group, such phenomenon was also seen in animal and childhood obesity, and can be ascribed to the “honeymoon obese phase” [6, 25, 26]. However, in group of OP, such decreased trend vanished, indicating asprosin seems to get involved in the periodontitis and obesity in a subtle way, with a combination of compensatory and anti-compensatory effect of balancing inflammation and systemic energy metabolism [27].

When we narrowed the focused population into patients with or without obesity, we found that adipokines, including ISM1 and asprosin were associated with periodontitis-parameters on the basis of CAL merely in obese population. This suggested that these two biomarkers might be potentially involved in the pathways of how periodontitis aggravated by obesity.

While this study provides valuable insights, several limitations should be acknowledged. First, as a cross-sectional design, it can only infer associations rather than establish causality. A longitudinal study with a larger sample size would be optimal to validate the role of these biomarkers in the obesity-periodontitis comorbidity.

Second, although we rigorously collected demographic, medical (using reliable tertiary hospital reports within one year), and lifestyle data (e.g., smoking/alcohol intake) following NHANES standards [28], recall bias remains inevitable, particularly for self-reported habits like smoking and alcohol consumption,. Nevertheless, we minimized this through standardized protocols.

Third, it is important to note that this study measured total MMP-8 rather than active MMP-8 (aMMP-8), the latter being a more precise biomarker for periodontitis as demonstrated in recent studies [29, 30]. Due to the lack of commercially available detection methods for aMMP-8, we focused on total MMP-8 in oral rinse fluid, which still aligns with prior evidence supporting its diagnostic potential for periodontitis [30–32]. Future studies should prioritize investigating aMMP-8 to clarify its role in this comorbidity.

## Conclusion

In conclusion, the circulating levels of the ISM1 and MMP-8 are significantly higher in patients with obesity and are associated with BMI. Moreover, we reveal that levels of ISM1 and MMP-8 in GCF could be utilized as potential assessing tool for grade of periodontitis. Non-invasive method of testing ISM1, MMP-8 and asprosin could be an effective tool for evaluating periodontitis in patients with obesity. All in all, ISM1, MMP-8 and asprosin might be defining biomarkers in periodontitis with obesity. Meanwhile, within the guarantee of biosafety and efficacy, the activation or inhibition of the signaling of specific cytokines/adipokines, by using adipokine-neutralizing antibodies or other methods to control the production and secretion of which, may contribute to prevent or treat the development of periodontitis with obesity.

## Supplementary Information

Supplementary Material 1.

## Data Availability

No datasets were generated or analysed during the current study.
