# Supplementary material for: Isthmin 1, matrix metalloproteinase 8 and asprosin as potential biomarkers in periodontitis with obesity
Source: BMC Oral Health. 2025 Oct 8;25:1551. doi: 10.1186/s12903-025-06868-5 (PMC12506396; doi:10.1186/s12903-025-06868-5)
Supplement: Supplementary file 1 — Supplementary Material 1. [file 12903_2025_6868_MOESM1_ESM.docx]

**Isthmin 1, Matrix Metalloproteinase 8 and Asprosin as Potential Biomarkers in Periodontitis with Obesity**

Yuwei Zhang^1, 2, 3^, Yue Jia^1, 2^, Shanmei Zhao^4^, Yanqi Wang^1, 2^, MaErWa MuLaTiHaJi^1, 2^, Xiao Luo^5*^, Ru Jia^1, 2, 4*^

1. Key Laboratory of Shaanxi Province for Craniofacial Precision Medicine Research, College of Stomatology, Xi’an Jiaotong University, Xi’an 710004, Shaanxi Province, China

2. Clinical Research Center of Shaanxi Province for Dental and Maxillofacial Diseases, Xi’an 710004, Shaanxi Province, China

3. Department of Biomedical Engineering, The Chinese University of Hong Kong, NT, Hong Kong SAR, 999077, P. R. China

4. Department of Digital Oral Implantology and Prosthodontics, College of Stomatology, Xi’an Jiaotong University, Xi’an 710004, Shaanxi Province, China

5. Department of Physiology and Pathophysiology, School of Basic Medical Sciences, Xi'an Jiaotong University Health Science Center, Xi'an 710061, Shaanxi, China

*Correspondence to*: Dr Ru Jia or Professor Xiao Luo, Key Laboratory of Shaanxi Province for Craniofacial Precision Medicine Research, College of Stomatology, Xi'an Jiaotong University, 98 Xiwu Road, Xi'an, Shaanxi 710004, P.R. China

E‑mail: [jiaru1987@mail.xjtu.edu.cn](mailto:jiaru1987@mail.xjtu.edu.cn)

E‑mail: [xluo@mail.xjtu.edu.cn](mailto:xluo@mail.xjtu.edu.cn)

Table S1. Questionnaires of Integrated Medical & Lifestyle Assessment Form.

Section 1: Demographics & Medical History

| **Category** | **Parameter** | **Response Options / Notes** |
| --- | --- | --- |
| Demographics | Respondent ID (SEQN) | ________ |
|  | Gender | ☐ Male ☐ Female |
|  | Age | ________ years |
|  | Height | ______ cm |
|  | Weight | ______ kg |
|  | BMI | ______ kg/m² |
| Medical History* | Hypertension | ☐ Yes ☐ No |
|  | Pulmonary Disease | ☐ Yes (Specify: ______) ☐ No |
|  | Renal Disease | ☐ Yes (Specify: ______) ☐ No |
|  | Metabolic Disease | ☐ Diabetes ☐ Dyslipidemia ☐ None |
|  | Cardiovascular Disease | ☐ Yes (Specify: ______) ☐ No |
|  | Immune System Disease | ☐ Yes (Specify: ______) ☐ No |
| Lifestyle Factors | Current Smoking Habit | ☐ Yes (Fill Section 2) ☐ No |
|  | Current Drinking Habit | ☐ Yes (Fill Section 3) ☐ No |
|  | Frequency of Brushing | ☐ More than once/day ☐ Not more than once/day |
|  | Physical Activity Level | ☐ Low ☐ Moderate ☐ High |

* **Note:** Patients are required to provide a high-reliability medical examination report from a Tier 3A hospital within the past year.

Section 2: Smoking Behavior Survey

| **Question** | **Response Options** |
| --- | --- |
| 1. Smoked ≥100 cigarettes in lifetime? | ☐ Yes ☐ No (If "No," skip to Section 3) |
| 2. Age started smoking regularly: | ______ years old |
| 3. Do you currently smoke cigarettes? | ☐ Yes ☐ No |
| 4. If quit, how long since quitting? | ______ [Number] |
| - Unit of time: | ☐ Days ☐ Weeks ☐ Months ☐ Years |
| 5. Age last smoked regularly: | ______ years old |
| 6. # cigarettes/day when quit: | ______ cigarettes/day |
| 7. How soon after waking do you smoke? | ☐ ≤5 min ☐ 6–30 min ☐ 31–60 min ☐ >60 min ☐ N/A (non-smoker) |
| 8. # days smoked in past 30 days: | ______ days |
| 9. Avg # cigarettes/day (past 30 days): | ______ cigarettes/day |
| 10. Avg # cigarettes/year (past 1 year): | ______ cigarettes/year |
| 11. Cigarette pack inspection (if applicable): | ☐ Pack shown ☐ Not shown ☐ N/A |

Section 3: Alcohol Consumption Survey

| **Question** | **Response Options** |
| --- | --- |
| 1. Consumed ≥12 alcohol drinks in past 12 months? | ☐ Yes ☐ No (Skip to Q3 if "No") |
| 2. Ever consumed ≥12 alcohol drinks in lifetime? | ☐ Yes ☐ No |
| 3. Frequency of alcohol consumption (past 12 months)? | ☐ Daily ☐ Weekly ☐ Monthly ☐ Yearly ☐ Never |
| - If weekly/monthly/yearly, specify days: | ______ days per ☐ Week ☐ Month ☐ Year |
| 4. Average # drinks per day (past 12 months)? | ______ drinks/day |
| 5. # days with heavy drinking (4/5+ drinks/day) in past 12 months? | ______ days |
| - Unit for heavy drinking days: | ☐ Days/Week ☐ Days/Month ☐ Days/Year |
| 6. Ever drank 4/5+ drinks daily? | ☐ Yes ☐ No |
| 7. # times consumed 4/5+ drinks within 2 hours (past 12 months)? | ______ times |

Table S2. *P* value of between two groups confirmed by LSD test or nonparametric test from Figure 1A to 1F.

| **Fig1A** | **NnP** | **OnP** | **NP** | **OP** |
| --- | --- | --- | --- | --- |
| **NnP** | - | - | - | - |
| **OnP** | **< 0.0001** | - | - | - |
| **NP** | 0.0808 | **< 0.0001** | - | - |
| **OP** | **< 0.0001** | 0.5071 | **< 0.0001** | - |
| **Fig1B** | | | | |
| **NnP** | - | - | - | - |
| **OnP** | 0.5385 | - | - | - |
| **NP** | 0.3250 | 0.7155 | - | - |
| **OP** | 0.9954 | 0.5426 | 0.3282 | - |
| **Fig1C** | | | | |
| **NnP** | - | - | - | - |
| **OnP** | **0.0069** | - | - | - |
| **NP** | 0.2144 | **0.0448** | - | - |
| **OP** | 0.2803 | 0.7126 | 0.6439 | - |
| **Fig1D** |  |  |  |  |
| **NnP** | - | - | - | - |
| **OnP** | **0.0015** | - | - | - |
| **NP** | 0.7709 | **0.0002** | - | - |
| **OP** | **0.0076** | 0.5826 | **0.0014** | - |
| **Fig1E** |  |  |  |  |
| **NnP** | - | - | - | - |
| **OnP** | 0.7529 | - | - | - |
| **NP** | 0.1318 | 0.1001 | - | - |
| **OP** | 0.2991 | 0.3273 | 0.7708 | - |
| **Fig1F** | | | | |
| **NnP** | - | - | - | - |
| **OnP** | **0.0259** | - | - | - |
| **NP** | **0.0293** | 0.5626 | - | - |
| **OP** | **0.0033** | 0.3280 | 0.8229 | - |

Table S3. *P* value of between two groups confirmed by LSD test or nonparametric test from Figure 2A to 2C

| **Fig2A** | **NnP** | **OnP** | **NP** | **OP** |
| --- | --- | --- | --- | --- |
| **NnP** | - | - | - | - |
| **OnP** | **0.0049** | - | - | - |
| **NP** | 0.6539 | **0.0004** | - | - |
| **OP** | **0.0103** | 0.8887 | **0.0013** | - |
|  | | | | |
| **NnP** | - | - | - | - |
| **OnP** | 0.0726 | - | - | - |
| **NP** | **0.0173** | 0.6109 | - | - |
| **OP** | **0.0339** | 0.5221 | 0.8269 | - |
|  | | | | |
| **NnP** | - | - | - | - |
| **OnP** | 0.5502 | - | - | - |
| **NP** | 0.3475 | 0.6619 | - | - |
| **OP** | 0.1179 | 0.4853 | 0.9179 | - |
| **Fig2B** |  |  |  |  |
| **NnP** | - | - | - | - |
| **OnP** | **0.0182** | - | - | - |
| **NP** | 0.6370 | **0.0479** | - | - |
| **OP** | **0.0003** | 0.2200 | **0.0031** | - |
|  |  |  |  |  |
| **NnP** | - | - | - | - |
| **OnP** | **0.0204** | - | - | - |
| **NP** | **0.0020** | 0.4729 | - | - |
| **OP** | **0.0001** | 0.2401 | 0.6710 | - |
|  | | | | |
| **NnP** | - | - | - | - |
| **OnP** | 0.3973 | - | - | - |
| **NP** | 0.1727 | 0.0741 | - | - |
| **OP** | **0.0443** | **0.0205** | 0.3988 | - |
| **Fig2C** | | | | |
| **NnP** | - | - | - | - |
| **OnP** | **0.0125** | - | - | - |
| **NP** | 0.9758 | **0.0015** | - | - |
| **OP** | 0.7515 | **0.0014** | 0.6339 | - |
|  |  |  |  |  |
| **NnP** | - | - | - | - |
| **OnP** | **< 0.0001** | - | - | - |
| **NP** | 0.9493 | **< 0.0001** | - | - |
| **OP** | 0.3946 | **0.0010** | 0.3912 | - |
|  |  |  |  |  |
| **NnP** | - | - | - | - |
| **OnP** | 0.3276 | - | - | - |
| **NP** | 0.6299 | 0.7507 | - | - |
| **OP** | 0.0992 | 0.4897 | 0.3649 | - |


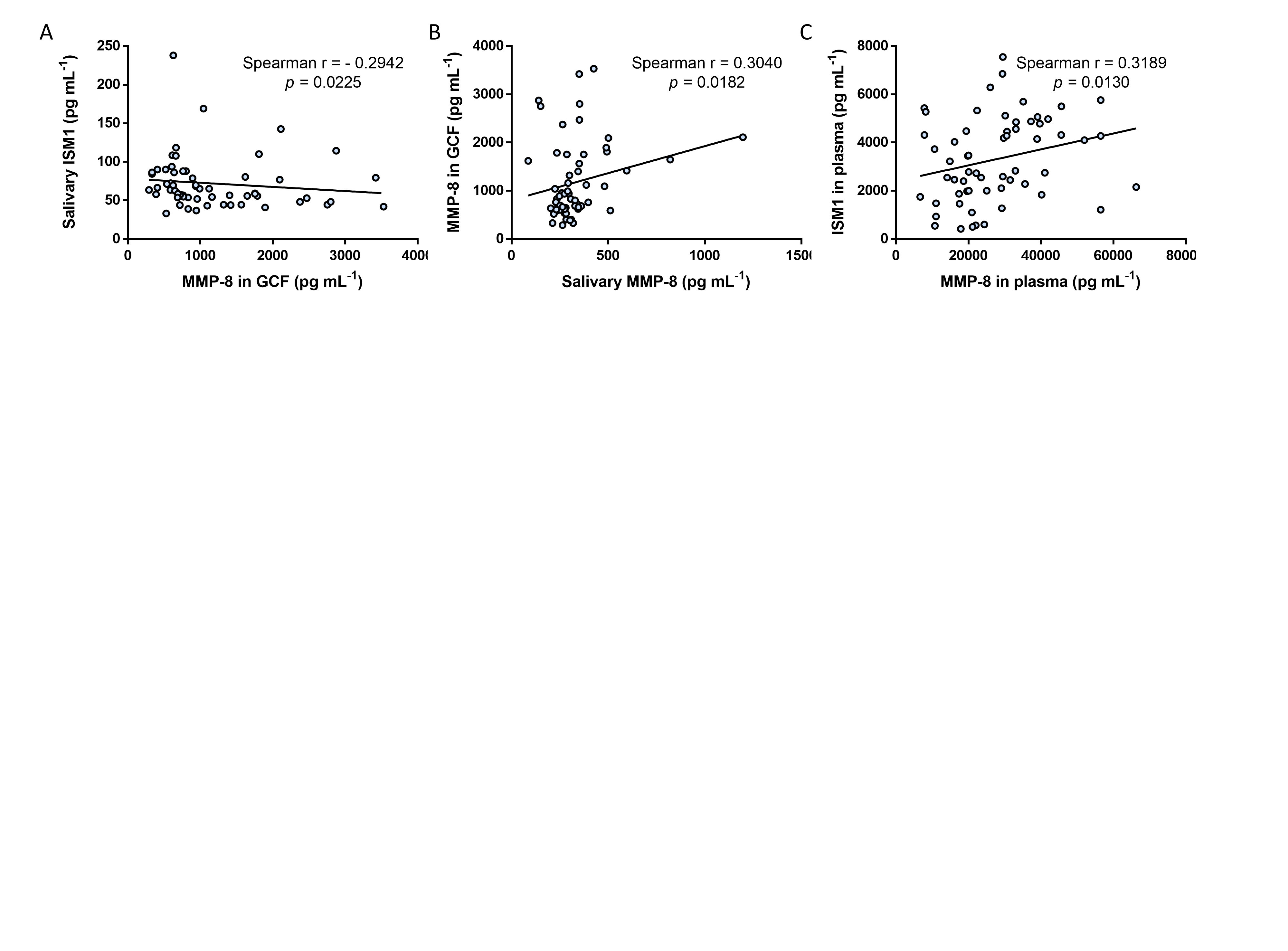


Figure S1. Correlation analysis of aimed biomarkers in whole population (n = 60), the Spearman correlation analysis was used as the statistical test.

Table S4. Negative binomial regression of factors influencing periodontal pocket depth in obese population.

| **Variables** | **β** | **Std. Error** | ***Wald*** | ***P*** | ***IRR*** | **95%*CI*** |
| --- | --- | --- | --- | --- | --- | --- |
| ISM1 in GCF | 0.049 | 0.0005 | 8.809 | 0.022* | 1.051 | 1.007,  1.096 |
| MMP-8 in GCF | 0.001 | 0.0004 | 5.570 | 0.018* | 1.001 | 1.000,  1.002 |
| Asprosin in GCF | 0.522 | 0.1899 | 7.547 | 0.006* | 1.685 | 1.161,  2.445 |
| BMI | 0.344 | 0.1647 | 4.372 | 0.037* | 1.411 | 1.022,  1.949 |

*Significant at *p* value <0.05.

Table S5. Negative binomial regression of factors influencing periodontal pocket depth in non-obese population.

| **Variables** | **β** | | **Std. Error** | ***Wald*** | ***P*** | ***IRR*** | **95%*CI*** |
| --- | --- | --- | --- | --- | --- | --- | --- |
| ISM1 in GCF | | 0.079 | 0.0271 | 8.580 | 0.003* | 1.083 | 1.027,  1.142 |
| MMP-8 in GCF | | 0.002 | 0.0005 | 8.211 | 0.004* | 1.002 | 1.000,  1.003 |
| Asprosin in GCF | | -0.414 | 0.4995 | 0.688 | 0.407 | 0.661 | 0.248,  1.759 |
| BMI | | 0.334 | 0.2659 | 1.573 | 0.210 | 1.396 | 0.829,  2.351 |

*Significant at *p* value <0.05.

Table S6. Negative binomial regression of factors influencing clinical attachment loss in obese population.

| **Variables** | **β** | | **Std. Error** | ***Wald*** | ***P*** | ***IRR*** | **95%*CI*** |
| --- | --- | --- | --- | --- | --- | --- | --- |
| ISM1 in GCF | | 0.051 | 0.0255 | 4.068 | 0.044* | 1.053 | 1.001,  1.107 |
| MMP-8 in GCF | | 0.001 | 0.0005 | 6.787 | 0.009* | 1.001 | 1.000,  1.002 |
| Asprosin in plasma | | 0.182 | 0.0682 | 7.136 | 0.008* | 1.200 | 1.050,  1.371 |
| Asprosin in GCF | | 0.592 | 0.2018 | 8.601 | 0.003* | 1.807 | 1.217,  2.684 |
| BMI | | 0.371 | 0.1732 | 4.594 | 0.032* | 1.449 | 1.032,  2.035 |

*Significant at *p* value <0.05.

Table S7. Negative binomial regression of factors influencing clinical attachment loss in non-obese population.

| **Variables** | **β** | | **Std. Error** | ***Wald*** | ***P*** | ***IRR*** | **95%*CI*** |
| --- | --- | --- | --- | --- | --- | --- | --- |
| ISM1 in GCF | | 0.040 | 0.0321 | 1.580 | 0.209 | 1.041 | 0.978,  1.109 |
| MMP-8 in GCF | | 0.003 | 0.0007 | 17.586 | 0.000* | 1.003 | 1.001,  1.004 |
| Asprosin in plasma | | -0.008 | 0.0269 | 0.079 | 0.779 | 0.992 | 0.941,  1.046 |
| Asprosin in GCF | | 0.586 | 0.4824 | 1.475 | 0.225 | 1.797 | 0.698,  4.625 |
| BMI | | 0.281 | 0.2123 | 1.747 | 0.186 | 1.324 | 0.873,  2.007 |

*Significant at *p* value <0.05.
